# Risks and benefits of ChatGPT in informing patients and families with rare kidney diseases: an explorative assessment by the European Rare Kidney Disease Reference Network (ERKNet)

**DOI:** 10.1007/s00467-025-06746-w

**Published:** 2025-04-16

**Authors:** Albertien M. van Eerde, Ana Teixeira, Flavia Galletti, Michal Maternik, Valentina Capone, Rik Westland, Jaap Mulder, Jan Halbritter, Thomas Osterholt, Valentina Neukel, Lutz T. Weber, Max C. Liebau, Franz Schaefer, Stefan Kohl

**Affiliations:** 1 Department of Genetics, University Medical Center, Utrecht, The Netherlands; 2 Division of Pediatric Nephrology, Centro Materno-Infantil Do Norte, Unidade Local de Saúde de Santo António, Porto, Portugal; 3 PKD International, Geneva, Switzerland; 4 Department of Pediatrics, Nephrology, Hypertension, Medical University of Gdansk, Gdansk, Poland; 5 Pediatric Nephrology, Dialysis and Transplant Unit, Fondazione IRCCS Ca’ Granda-Ospedale Maggiore Policlinico, Milan, Italy; 6 Department of Pediatric Nephrology, Emma Children’S Hospital - Amsterdam UMC, Location University of Amsterdam, Amsterdam, The Netherlands; 7 Department of Pediatrics, Division of Pediatric Nephrology, Sophia Children’s Hospital, Erasmus MC, Rotterdam, The Netherlands; 8 Department of Pediatrics, Division of Pediatric Nephrology, Willem-Alexander Children’S Hospital, Leiden University Medical Center, Leiden, The Netherlands; 9 Department of Nephrology and Medical Intensive Care, Charité Universitätsmedizin Berlin, Berlin, Germany; 10 Department II of Internal Medicine and Center for Molecular Medicine Cologne, Faculty of Medicine, University Hospital of Cologne, University of Cologne, Cologne, Germany; 11 Division of Pediatric Nephrology, Center for Pediatrics and Adolescent Medicine, University Hospital Heidelberg, Heidelberg, Germany; 12 Department of Pediatrics, Faculty of Medicine, University Hospital Cologne and University of Cologne, Cologne, Germany; 13 Center for Rare Diseases, University Hospital Cologne and Medical Faculty, University of Cologne, Cologne, Germany; 14 Center for Molecular Medicine Cologne, Faculty of Medicine, University of Cologne, University Hospital Cologne, Cologne, Germany

**Keywords:** Rare disease, Rare kidney disease, ChatGPT, Artificial intelligence, AI, Patient education, Genetic testing, Patient advocacy, Large language model

## Abstract

**Background:**

Rare diseases affect fewer than 1 in 2000 individuals, but approximately 150 rare kidney diseases account for about 10% of the chronic kidney disease (CKD) population, impacting millions across Europe and globally. The scarcity of medical experts for these conditions results in an unmet need for accurate and helpful patient information. Large language models like ChatGPT may offer a technological solution to assist medical professionals in educating patients and improving doctor-patient communication. We hypothesized that ChatGPT could provide accurate responses to frequently asked basic questions from patients with rare kidney diseases.

**Methods:**

Medical professionals and members of European Patient Advocacy Groups (ePAGs) affiliated with the European Rare Kidney Disease Reference Network (ERKNet) simulated patient-ChatGPT interactions using a Microsoft forms questionnaire and ChatGPT 3.5 and 4.0. Participants selected any rare kidney disease for a structured conversation with ChatGPT 3.5 or 4.0. Responses were evaluated for accuracy and helpfulness.

**Results:**

Forty-six ERKNet experts and 12 ePAGs from 13 European countries participated in this study. ChatGPT provided scientifically accurate and helpful information on 28 randomly selected rare kidney diseases, including prognostic information and genetic testing guidance. Participants expressed neutral positions regarding ChatGPT’s recommendations on alternative treatments, second opinions, and other information sources. While ChatGPT generally was perceived as helpful and empathetic, concerns about patient safety persisted.

**Conclusions:**

ChatGPT exhibited substantial potential in addressing patient inquiries regarding rare kidney diseases in a real-world context. While it demonstrated resilience against misinformation in this application, careful human oversight remains essential and indispensable.

**Graphical abstract:**

**Figure:**
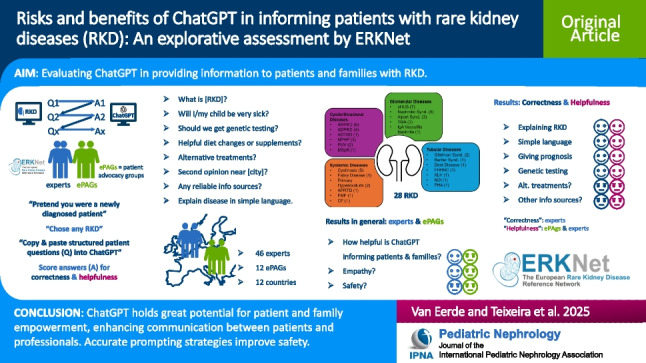
A higher resolution version of the Graphical abstract is available as Supplementary information

**Supplementary Information:**

The online version contains supplementary material available at 10.1007/s00467-025-06746-w.

## Introduction

Rare kidney diseases (RKDs) collectively represent a significant subset of chronic kidney disease (CKD), accounting for approximately 5–10% of CKD cases in adults and almost all cases of CKD in children in Europe [1, 2]. Although each individual RKD affects fewer than 1 in 2000 individuals, the cumulative impact is substantial, involving about two million patients across Europe and many more globally [3, 4]. These conditions encompass a broad spectrum of genetic, structural, and functional disorders, which manifest in pediatric and adult populations [5]. The diversity and complexity of RKDs present significant challenges not only for diagnosis and treatment but also for patient education, communication, and support [6].

The European Rare Kidney Disease Reference Network (ERKNet) was established to address these challenges by providing a collaborative platform for clinical care, research, and education across Europe. ERKNet unites specialists in pediatric and adult nephrology, human genetics, pathology, and patient advocacy to enhance awareness, diagnosis, and treatment of RKD. Despite these efforts, there remains a considerable information deficit among patients and their families, particularly regarding disease management, prognosis, and available treatments. The resulting information deficit experienced by patients and their families often prompts them to independently seek information on the Internet, including through social media [7]. It is well-established that medical information found on social media and non-professional platforms carries a significant risk of misinformation, which can potentially be harmful [8].

The advent of artificial intelligence (AI) and large language models (LLMs) such as ChatGPT (Generative Pre-trained Transformer) offer a promising tool to bridge this information gap and in this way reduce inequities in access to information [9]. ChatGPT, developed by OpenAI, is pre-trained on extensive text data, fine-tuned with model-specific tools, and designed to provide responses to user queries in a conversational manner [9]. It is the most popular and most frequently used LLM today. While the potential of AI-driven models in patient education is widely recognized, their application in providing reliable medical information to patients, especially in the context of rare diseases, is still under investigation [10–12].

Previous studies have examined the use of ChatGPT in various medical contexts, highlighting its capacity to produce coherent, contextually appropriate responses and even demonstrating its ability to pass the United States Medical Licensing Examination (USMLE) [13, 14]. However, concerns regarding the accuracy, relevance, and safety of the information provided by ChatGPT persist. There is a need to evaluate whether ChatGPT can reliably address the specific needs of patients with rare diseases, where the availability of specialized knowledge is crucial.

This study aims to assess the performance of ChatGPT 3.5 and 4.0, as the most frequently used LLMs, in providing accurate and helpful information to patients with rare kidney diseases in a real-world setting. By engaging ERKNet experts and members of European Patient Advocacy Groups (ePAGs) in simulated patient-ChatGPT interactions, we evaluate the model’s ability to deliver information that aligns with current clinical knowledge and meets the needs of patients and their families.

## Methods

### Ethical approval

Ethical approval for this survey study was obtained from the University of Cologne Institutional Review Board (IRB approval number 24–1072).

### Recruitment of participants

Professional participants at the 8th annual meeting of the European Rare Kidney Disease Network (ERKNet), held in Venice, Italy, in 2024, were invited to take part in this study. Eligible participants included “ERKNet experts,” such as pediatric nephrologists, adult nephrologists, human geneticists, or basic researchers with experience in treating patients with rare kidney diseases, as well as ePAGs involved in rare kidney diseases. Participation was voluntary and anonymous. All participants were required to sign up for either a free ChatGPT 3.5 or ChatGPT 4.0 account, if they had not done so previously.

### Survey development

The ChatGPT-patient interactions were carefully developed based on clinical experience of ERKNet experts and ePAGs caring for patients with RKD. Survey questions and instructions for interacting with ChatGPT were compiled in a Microsoft Forms document (Supplemental Table 1). To avoid responses influenced by previous chats on participants’ ChatGPT accounts, the survey began with the prompt: “For this conversation, please treat me as a non-medical user with an average education.” At the start of the conversation, participants were instructed to select any rare kidney disease within their field of expertise, thereby minimizing bias in selection of RKD (Supplemental Table 1). Participants could adopt either a pediatric perspective (“my child has”) or an adult perspective (“I have”).

All follow-up questions pertained to the initially selected condition.

Disease-related questions, which had to be copied and pasted into ChatGPT by the participants, included the following:

1. I have/My child has [name of rare kidney disease]. Please explain what that is.
2. I am worried. Am I/Is my child going to be very sick?
3. Should I/we get genetic testing?
4. Are there any helpful dietary modifications or supplements?
5. Are there any alternative treatments?
6. Where can I find a doctor for a second opinion close to [name of a familiar city]?
7. Are there any other reliable information sources?
8. What disease does our child have? Explain in plain language.

Participants scored each ChatGPT response to these questions based on two criteria: scientific correctness (Does the provided response align with current clinical knowledge and scientific understanding?) and helpfulness (In your opinion, how helpful would this response be for a patient or their family?). In agreement with our real-word approach, an ordinal scale was used for scoring, with the following values: 1 (extremely negative), 2 (negative), 3 (neutral), 4 (positive), and 5 (extremely positive). Median scores of 4 (“positive”) and 5 (“extremely positive”) indicate broad agreement among our participants with ChatGPT. A median score of > 2 and < 4 was considered neutral. A median score of 1 (“extremely negative”) and 2 (“negative”) indicate broad disagreement with ChatGPT.

ERKNet experts were requested to formulate one or two “expert-level questions” to their selected condition to challenge ChatGPT’s capabilities. In contrast, ePAGs were asked to challenge ChatGPT by presenting a hypothetical critical or emotional patient scenario and seeking its assistance. Additional questions were included to collect information on the version of ChatGPT used (3.5 or 4.0), the participant’s age group (< 30, 30–50, and > 50 years), field of expertise (free text), role (ERKNet expert or ePAG), and prior experience with ChatGPT (for the first time, for fun, for assistance in scientific writing, or for everyday tasks such as emails or medical reports).

### Use of ChatGPT for manuscript preparation

In the development of this manuscript, the authors utilized ChatGPT to refine the language, similarly to employing a native-speaking editor. Following the use of this tool, the authors thoroughly reviewed and edited the content as necessary and assume full responsibility for the final version of the publication.

## Results

A total of 54 participants (42 ERKNet experts and 12 ePAG representatives) provided valid responses that were included in this analysis. Responses from four ERKNet experts were excluded due to the use of imprecise disease terminology (e.g., “polycystic kidney disease” rather than specifying ADPKD or ARPKD). Among the 54 participants, 34 were aged 30–50 years, and 20 were over 50 years old. Participants were from various countries, including Germany (*n* = 13), the Netherlands (*n* = 8), Italy (*n* = 6), Spain (*n* = 5), Belgium (*n* = 2), Poland (*n* = 2), Sweden (*n* = 2), the UK (*n* = 2), and one participant each from the Czech Republic, France, Ireland, Malta, Romania, and Slovenia, while 8 participants did not wish to reveal their country of origin (Supplemental Fig. 1). In terms of professional background, 32 participants identified as pediatric nephrologists, 7 as adult nephrologists, and 3 as pathologists. Regarding prior experience with ChatGPT, 16 participants reported using it for the first time, 19 had used it only “for fun,” and 19 had also used it for work-related tasks.

**Fig. 1:**
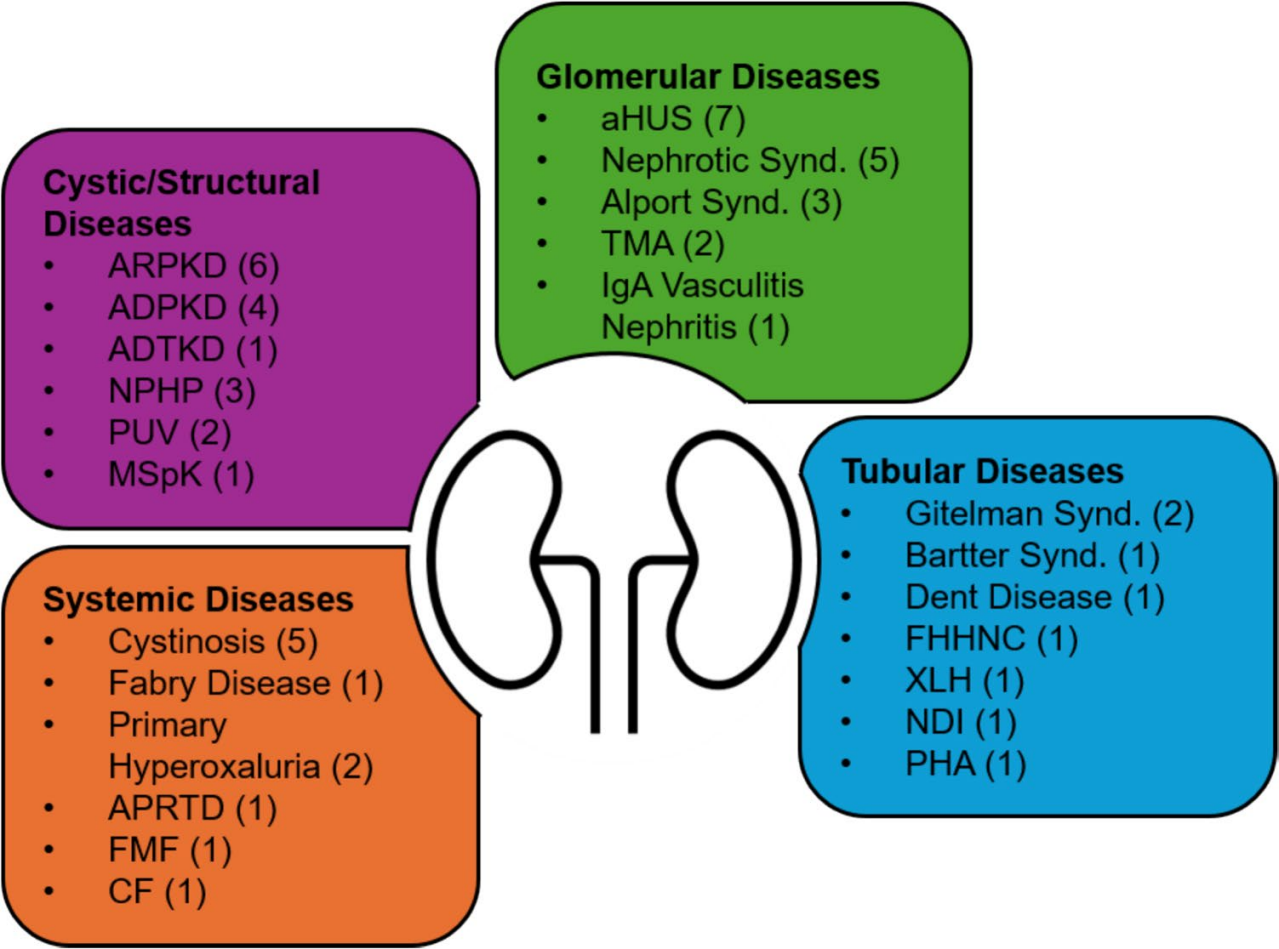
Twenty-eight rare kidney diseases selected by 54 ERKNet experts and ePAGs. ADPKD, autosomal dominant polycystic kidney disease; ADTKD, autosomal dominant tubulointerstitial kidney disease; aHUS, atypical hemolytic uremic syndrome; APRTD, adenine phosphoribosyltransferase deficiency; ARPKD, autosomal recessive polycystic kidney disease; CF, cystic fibrosis; FHHNC, familial primary hypomagnesemia with hypercalciuria and nephrocalcinosis; FMF, familial mediterranean fever; MSpK, medullary sponge kidney; NDI, nephrogenic diabetes insipidus; NPHP, nephronophthisis; PHA, pseudohypoaldosteronism; PUV, posterior urethral valves; TMA, thrombotic microangiopathy; XLH, X-linked hypophosphatemia

The 54 participants selected a total of 28 different rare kidney diseases. The most frequently selected conditions included atypical hemolytic uremic syndrome (aHUS) (*n* = 6), autosomal recessive polycystic kidney disease (ARPKD) (*n* = 6), cystinosis (*n* = 5), nephrotic syndrome (*n* = 5), autosomal dominant polycystic kidney disease (ADPKD) (*n* = 4), nephronophthisis (*n* = 3), Alport syndrome (*n* = 3), Gitelman syndrome (*n* = 2), posterior urethral valves (*n* = 2), primary hyperoxaluria (*n* = 2), and thrombotic microangiopathy (*n* = 2) (Fig. 1).

For evaluating whether ChatGPT’s responses to various survey questions align with current scientific knowledge, we considered only the scores from 42 ERKNet experts (Table 1). For evaluating whether ChatGPT’s responses to various survey questions are helpful for patients and families, we considered scores from ERKNet experts and ePAGs (Table 1).

**Table 1:** Evaluation of the “scientific correctness” and “helpfulness” of ChatGPT responses

Survey copy and paste prompt for ChatGPT	Score, median (1st and 3rd quartile)	Result	Score, median (1st and 3rd quartile)	Result
	Correctness (42 ERKNet experts)		Helpfulness (42 ERKNet experts and 12 ePAGs)	
My child has [name of rare kidney disease]. Please explain what that is	4 (4, 4)	Positive	4 (3, 5)	Positive
I am worried. Is my child going to be very sick?	4 (3.25, 5)	Positive	4 (3, 5)	Positive
Should we get genetic testing?	4.5 (4, 5)	Positive	4 (4, 5)	Positive
Are there any helpful dietary modifications or supplements?	4 (3, 4)	Positive	4 (3, 5)	Positive
Are there any alternative treatments?	3 (2, 4)	Neutral	3 (2, 4)	Neutral
Where can I find a doctor for a second opinion close to [name of a familiar city]?	n/a	n/a	3 (2, 4)	Neutral
Are there any other reliable information sources?	n/a	n/a	3 (2, 4)	Neutral
What disease does our child have? Explain in plain language	n/a	n/a	4 (3, 4)	Positive
ERKNet expert question 1 (*n* = 54)	4 (3, 5)	Positive	n/a	n/a
ERKNet expert question 2 (*n* = 23)	3 (3, 4.5)	Neutral	n/a	n/a

*n/a*, not applicable

Our findings demonstrate that both ChatGPT 3.5 and 4.0 provide explanations of rare kidney diseases to patients and families that are consistent with scientific understanding and are considered helpful for patients and families (Table 1). Additionally, the prognostic information about the underlying disease and guidance on the decision whether to obtain genetic testing are presented accurately and in a helpful manner (Table 1). However, ERKNet experts and ePAGs expressed concerns about ChatGPT’s responses to questions related to alternative treatments, options for seeking a second opinion in various European cities, and recommendations for other reliable information sources (Table 1). ChatGPT’s ability to explain diseases in plain language was considered accurate and helpful (Table 1).

Responses to random expert-level questions (Supplemental Table 2) from participating experts were generally accurate (Table 1). In one instance, an expert remarked that the response in context of nephrogenic diabetes insipidus, “seek medical attention if signs of dehydration, such as dry mouth, sunken eyes, or decreased urination occur,” was “inappropriate” and “potentially harmful,” as patients should seek medical attention at an earlier stage. ChatGPT’s responses to the “emotional challenges” presented by ePAGs received a median score of 3, with a relatively broad interquartile range of 2.25, indicating mixed satisfaction (Table 2).

**Table 2:** General aspects of ChatGPT responses

Survey question to ERKNet experts and ePAGs on general aspects of ChatGPT responses	Score by ERKNet experts, median (1st and 3rd quartile)	Result	Score by ePAGs, median (1st and 3rd quartile)	Result
In your opinion, how helpful could ChatGPT be for patients with rare diseases?	4 (3, 4)	Positive	3.5 (2.75, 4)	Neutral
In your opinion, are the responses by ChatGPT empathic?	4 (3, 4)	Positive	3.5 (2, 4)	Neutral
Based on your experience in this survey, how safe may ChatGPT be for patients and families?	3 (3, 4)	Neutral	3 (2.75, 4)	Neutral
ePAGs emotional challenge scenario	n/a	n/a	3 (2, 4.25)	Neutral

ERKNet experts and ePAGs generally agreed that ChatGPT is helpful and empathetic (Table 2). However, concerns about safety of this new technology in general remained, as reflected by a neutral score on that specific question (Table 2).

About half of the participants shared comments on one or more survey questions. Overall, we received 50 comments from 36/54 participants comprising mixed feedback on specific ChatGPT responses and general matters (Supplemental Table 3). Many appreciated ChatGPT’s clear and readable responses (“I am a bit surprised. The answers are way better than I thought they would be”). However, several participants criticized the responses for being too general, lacking specific medical details, and sometimes offering information that was not directly relevant to the condition in question (“Very generic answer. Uses terms such as ‘reabsorption,’ which is probably meaningless or even confusing without explanation”). Concerns were raised about ChatGPT’s suggestions of alternative treatments, such as herbal remedies and complementary medicine, which were seen as potentially misleading or unsafe (“Recommends ginger and licorice! As well as any other quackery around, such as ‘Mind–body-techniques’”). Participants also observed that ChatGPT often recommended consulting a healthcare provider, which was perceived positively. However, the exclusion of important resources, such as the National Institutes of Health (NIH), the European Medicines Agency (EMA), the International Pediatric Nephrology Association (IPNA), the Pediatric Nephrology Research Consortium (PNRC), Nephcure, and the European Rare Kidney Disease Reference Network (ERKNet), was considered a notable limitation. Additionally, the language used in responses was sometimes considered too technical or abstract for the average patient, and some answers appeared to be more US-centric rather than tailored to a European audience (“ERKNet and ESPN as well as ESPU are missing. ChatGPT suggests rather US organizations like Mayo Clinic and National Kidney Foundation”

## Discussion

With the help of 42 ERKNet experts and 12 ePAGs, we assessed the potential risks and benefits of ChatGPT as an information source for patients and families with rare kidney diseases. Consistent with previous studies evaluating ChatGPT’s effectiveness in patient education for common conditions such as prostate cancer, amblyopia, or systemic lupus erythematosus, our international survey participants reported that ChatGPT provided accurate and helpful responses to both basic and advanced questions across a wide range of rare kidney diseases. Our survey highlights the great potential of ChatGPT in efficiently compiling and presenting information on a wide array of rare medical conditions, while tailoring it to address specific individual queries. Importantly, we did not encounter any responses from ChatGPT that were entirely incorrect or, more critically, immediately dangerous for patients. However, we did encounter responses that were somewhat vague, confusing, arbitrary, not directly relevant to the topic, and ultimately not helpful.

Some experts raised concerns that questions about alternative treatment options or dietary modifications elicited responses from ChatGPT that were somewhat evasive, potentially undermining the primary emphasis on evidence-based therapy. Questions regarding other resources and medical centers for a second opinion often resulted in US-centric responses, which were frequently deemed unhelpful for mostly European users. However, with ongoing advancements in LLM technology, these issues are likely to be addressed in future versions or medical-content specific GPTs. Considering the undefined nature of training datasets for AI-powered public chatbots, including ChatGPT, and the “black box” nature of AI-driven decision-making, the authors argue that it is premature to deem this technology suitable for patient education without human oversight.

LLM-generated “hallucinations,” which can be described from a human perspective as inaccurate or inappropriate responses, as we encountered on the subjects of “alternative treatments” and “second opinions,” are a potentially dangerous phenomenon [15]. Notably, ChatGPT demonstrates greater resilience to hallucinations compared to other LLMs [15]. In this study, our questions regarding alternative treatment methods likely prompted ChatGPT to give hallucinatory responses. It is concerning that large language models (LLMs) do not clearly indicate when their knowledge is insufficient to provide a robust, fact-based response and instead present disputed viewpoints in an overly eloquent manner or, in some instances, relay incorrect information. For instance, when repeatedly asked, “Who wrote the book *From Fish to Philosopher*?” (the correct answer being Homer W. Smith, 1953), ChatGPT 3.5, at the time this study was conducted, provided numerous names with coherent explanations, though all were incorrect. This underscores the need for caution: while ChatGPT has the potential to enhance patient empowerment by providing accessible information, it is essential to remind patients that this information could be incorrect and that health-related decisions should always be discussed with their healthcare providers.

This study has several limitations. Firstly, while participants were asked to use an initial prompt to avoid influence from prior chats, we chose not to control ChatGPT metrics, as this study was designed as a test balloon for real-world ChatGPT-patient interactions. Additionally, the study focused only on ChatGPT versions 3.5 and 4.0, excluding newer models such as 4o and LLM from other companies. Another aspect that we did not assess, as our study strictly adhered to using the English language to ensure consistency in evaluation, is the multilingual capabilities of ChatGPT in the context of rare kidney disease education. Future research could explore how ChatGPT performs in different languages, whether translation affects the accuracy and clarity of responses, and how cultural nuances influence medical advice.

Despite the study’s limitations and the need for caution, the authors conclude that ChatGPT represents a significant technological advancement with a lower susceptibility to misinformation and manipulation by individual content creators compared to social media platforms. To enhance patient-ChatGPT interactions, a viable approach is the implementation of carefully crafted prompting strategies. Therefore, based on our experience with this survey, we have developed a set of prompting expressions designed to elicit individualized, accurate, and evidence-based responses from ChatGPT, thereby enhancing the provision of safe and reliable medical information (Box 1). These suggestions could serve as a foundation for developing patient education materials on the safe and effective use of AI, rather than leaving patients to navigate these tools on their own.

**Box 1:** Useful prompting expressions for patient-ChatGPT interaction

**Obtaining trustworthy information**
• “Can you provide me with trustworthy medical information about [disease/condition] from reliable sources like WHO or national health organizations?”
• “What are the scientifically proven treatments for [disease/condition]?”
• “What treatments for [disease] should I avoid because they lack scientific evidence?”
**Adapting language according to education level**
• For patients with higher education: “Can you explain the current research and scientific consensus on the treatment options for [disease]?”
• For patients with lower education: “Can you explain in simple terms what causes [disease] and how it can be treated?”
**Regional context**
• “Please take into consideration that I live in [city/country].”

To mitigate the limitations posed by uncontrollable ChatGPT pre-training dataset sources, ERKNet has launched a project leveraging LLM technology fine-tuned on carefully curated datasets, specifically tailored to rare kidney diseases. Furthermore, exploring the integration of a “human-in-the-loop” interface for potentially dangerous situations could be a valuable approach to mitigating the limitations of AI-driven systems.

The urgent need to disseminate genetic and RKD knowledge among nephrology care providers was recently underscored by KDIGO [16]. In this context, expert-trained AI models hold great promise also in assisting physicians, particularly in counseling for ultrarare diseases, where most caregivers lack personal experience.

Ultimately, ChatGPT and similar models have the potential to serve as valuable tools for both clinicians and patients by assisting in the consolidation of disease-related knowledge. This could free up time in fast-paced clinical settings for more efficient and productive interactions between expert doctors and informed patients, thereby enhancing the overall quality of healthcare.

## Supplementary Information

Below is the link to the electronic supplementary material.

Graphical abstract (pptx 380 KB)

Supplementary file2 (docx 1.80 MB)

Supplementary file3 (XLSX 42 KB)

## Data Availability

All source data is available in supplementary file 3.
