# Supplementary material for: Risks and benefits of ChatGPT in informing patients and families with rare kidney diseases: an explorative assessment by the European Rare Kidney Disease Reference Network (ERKNet)
Source: Pediatr Nephrol. 2025 Apr 16;40(9):2899–905. doi: 10.1007/s00467-025-06746-w (PMC12296756; doi:10.1007/s00467-025-06746-w)
Supplement: Supplementary file 1 — Graphical abstract (pptx 380 KB) [file 467_2025_6746_MOESM1_ESM.pptx]

## Slide 1
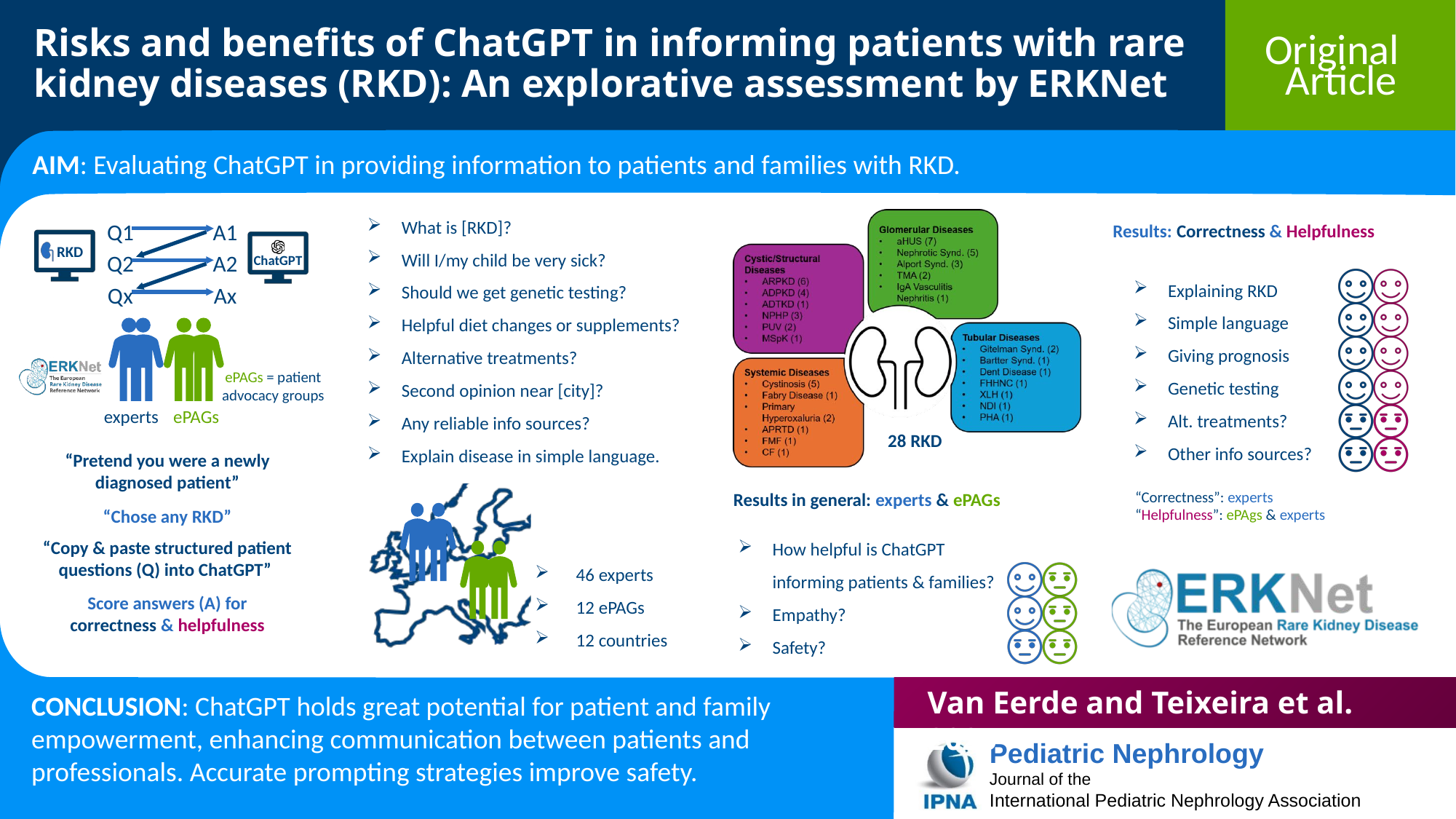

Risks and benefits of ChatGPT in informing patients with rare kidney diseases (RKD): An explorative assessment by ERKNet
AIM: Evaluating ChatGPT in providing information to patients and families with RKD.
What is [RKD]?
Will I/my child be very sick?
Should we get genetic testing?
Helpful diet changes or supplements?
Alternative treatments?
Second opinion near [city]?
Any reliable info sources?
Explain disease in simple language.
Q1
A1
ChatGPT
RKD
Q2
A2
Qx
Ax
ePAGs = patient advocacy groups
experts
ePAGs
“Pretend you were a newly diagnosed patient”
“Chose any RKD”
“Copy & paste structured patient questions (Q) into ChatGPT”
Score answers (A) for
correctness & helpfulness
Results: Correctness & Helpfulness
Explaining RKD
Simple language
Giving prognosis
Genetic testing
Alt. treatments?
Other info sources?
28 RKD
“Correctness”: experts
“Helpfulness”: ePAgs & experts
Results in general: experts & ePAGs
How helpful is ChatGPT informing patients & families?
Empathy?
Safety?
46 experts
12 ePAGs
12 countries
Van Eerde and Teixeira et al. 2025
CONCLUSION: ChatGPT holds great potential for patient and family empowerment, enhancing communication between patients and professionals. Accurate prompting strategies improve safety.
