# Supplementary material for: Risks and benefits of ChatGPT in informing patients and families with rare kidney diseases: an explorative assessment by the European Rare Kidney Disease Reference Network (ERKNet)
Source: Pediatr Nephrol. 2025 Apr 16;40(9):2899–905. doi: 10.1007/s00467-025-06746-w (PMC12296756; doi:10.1007/s00467-025-06746-w)
Supplement: Supplementary file 2 — Supplementary file2 (docx 1.80 MB) [file 467_2025_6746_MOESM2_ESM.docx]

**Risks and benefits of ChatGPT in informing patients and families with rare kidney diseases: An explorative assessment by the European Rare Kidney Disease Reference Network (ERKNet)**

**Supplemental material**

Supplemental Table 1: Transcript of Online Survey (Microsoft forms)

| **Survey: Can ChatGPT Aid in Informing Patients with Rare Diseases?**  Dear Patient Advocates and Rare Disease Experts,  Patients with rare diseases, along with their families and friends, often seek additional information about their conditions online or via social media. When they don't fully understand medical advice or have follow-up questions, they may turn to tools like ChatGPT, an AI-driven chatbot, for assistance. However, it is crucial to assess how well-informed, helpful, and safe ChatGPT is in providing information in this context.  We kindly request your participation in this anonymous survey to evaluate ChatGPT's effectiveness as a resource for stakeholders involved in rare diseases.  **Estimated time to complete the survey: 15 minutes**  Thank you for your invaluable cooperation!  **To begin:**   1. Visit https://openai.com/chatgpt. 2. If you already have a ChatGPT account, please log in and prepare to start a conversation. 3. If you don't have an account, click "Try ChatGPT" in the upper right corner and register to access the free version.   **Study Lead:**  PD Dr. med. Stefan Kohl  University Hospital of Cologne, Germany  Email: Stefan.kohl@uk-koeln.de |
| --- |
| 1  ChatGPT 4.0 You are using...  ChatGPT 3.5 "free version"  ChatGPT 4.0 |
| Pick a rare disease...  Please, start the structured conversation. Copy & paste the following sentence into ChatGPT: ***My child has [replace this by any rare disease that you are familiar with]. Please explain what that is.***  2  Which rare disease did you choose?    3  Does the provided response align with current clinical knowledge and scientific understanding?  1  2  3  4  5  4  How useful do you think this answer would be for a patient or a family just diagnosed with this condition?  1  2  3  4  5 |
| Feeling fear  5  Please, copy & paste the following sentence into ChatGPT: ***I am worried. Is my child going to be very sick?***  Does the provided response align with current clinical knowledge and scientific understanding?  1  2  3  4  5  6  In your opinion, how helpful would this response be for a patient or the family?  1  2  3  4  5 |
| Genetic testing  7  Please, copy & paste the following sentence into ChatGPT: ***Should we get genetic testing?***  Does the provided response align with current clinical knowledge and scientific understanding?  1  2  3  4  5  8  In your opinion, how helpful would this response be for a patient or the family?  1  2  3  4  5 |
| Dietary modifications  9  Please, copy & paste the following sentence into ChatGPT: ***Are there any helpful dietary modifications or supplements?***  Does the provided response align with current clinical knowledge and scientific understanding?  1  2  3  4  5  10  In your opinion, how helpful would this response be for a patient or the family?  1  2  3  4  5 |
| Complementary medicine  11  Please, copy & paste the following sentence into ChatGPT: ***Are there any alternative treatments?***  Does the provided response align with current clinical knowledge and scientific understanding?  1  2  3  4  5  12  In your opinion, how helpful would this response be for a patient or the family?  1  2  3  4  5 |
| Additional resources  13  Please, copy & paste the following sentence into ChatGPT: ***Where can I find a doctor for a second opinion close to [name of a familiar city]?***  In your opinion, how helpful would this response be for a patient or the family?  1  2  3  4  5  14  Please, copy & paste the following sentence into ChatGPT: ***Are there any other reliable information sources?***  In your opinion, how helpful would this response be for a patient or the family?  1  2  3  4  5 |
| Overall experience with ChatGPT  15  In your opinion, how helpful could ChatGPT be for patients with rare diseases?  1  2  3  4  5  16  In your opinion, are the responses by ChatGPT empathic?  1  2  3  4  5  17  Based on your experience in this survey, how safe may ChatGPT be for patients and families?  1  2  3  4  5 |
| Role in healthcare  18  Optional: What is your institution or your city?    19  You are...  ePAG  doctor / rare disease expert |
| Rare disease expert questionaire  20  What is your area of expertise or specialty?    21  Please formulate an expert-level question related to your condition to test ChatGPT's capabilities.  Please type your question below and then copy & paste it into ChatGPT.   What is your expert question?    22  Does the provided response align with current clinical knowledge and scientific understanding?  1  2  3  4  5  23  If you'd like (and it could be interesting), what would your second expert-level question be?    24  Does the provided response align with current clinical knowledge and scientific understanding?  1  2  3  4  5 |
| ePAG representatives "Emotional Challenge" questionnaire  20  Could you please present a hypothetical critical or emotional scenario to ChatGPT and ask for help?   Please type your scenario below and then copy & paste it into ChatGPT.    21  Do you believe the response is appropriate and helpful?  1  2  3  4  5 |
| Explaining rare diseases in plain language  25  Please, copy & paste the following sentence into ChatGPT: ***What disease do I have? Explain in plain language.***  In your opinion, how helpful would this response be for a patient or the family?  1  2  3  4  5 |
| Your relationship with ChatGTP  26  You have used ChatGPT...  ...for the first time  ...for fun  ...for assistance in scientific writing  ...for everyday tasks like emails or medical reports |
| Share your ChatGPT chat with us  27  Optional but helpful: You can share your survey chat on ChatGPT using the "arrow pointing up" symbol located in the upper right corner of the ChatGPT window. Please, paste the link below. Thank you!   |

Supplemental Table 2:

| **Unedited ERKNet experts challenges for ChatGPT** |
| --- |
| What is the risk for recurrence of disease after kidney transplantation? |
| How do variants in PKHD1 affect the clinical course of ARPKD? |
| Is bilateral nephrectomy in early life mandatory? |
| How my child should be followed? |
| What is the evidence for treatment with allopurinol or febuxostat in children with APRT? |
| In ADPKD, which environmental and genetic factors associated with phenotype variability? |
| My son has post renal transplant bk virus nephropathy should I be worried |
| To which extent do you believe that individuals with heterozygous variants in COL4A3 or COL4A4 have a real disease? |
| How would you assess the safety of the new drug Lumasiran? |
| How do i differentiate between those with rapid or slow disease progression in ADPKD? |
| Is burosumab superior than conventional treatment in XLH patients? |
| Should we do a nephrectomy in ARPKD? |
| What is the role for mTOR inhibitors in the treatment of cystinosis? |
| What are the known genes associated with ARPKD? |
| Define the latest translational therapies for fabry disease |
| For how long can my child stay without drinking? |
| What would be the pathophysiological possibilities, if genetic testing in aHUS is negative? |
| The relationship of MEST-C score with patient's nutritional status |
| How often chronic kidney disease occurs in children with PUV? |
| Who may indicate genetic testing? |
| Which are the genetic modifying factors of Alport syndrome? |
| Is prednisolon the right medicine to treat FSGS? |
| What are the benefits and disadvantages of tolvaptan |
| In aHUS with anti CFH antibodies shoud we use immunosuppressant theraphy and plasma exchange or Eculizunab as a complement blocking theraphy? |
| Does the clinical course of liver disease in ARPKD parallels the kidney disease course? |
| When to use rituximab in child with nephrotic syndrome and how many doses should be given? |
| What are the benefits of exchanging treatment from eculizumab to ravulizumab in an adolescent? |
| Does the disease affect other organs beyond the kidneys? |
| Will my child need dialysis? |
| what syndromal ciliopathies overlap with nephronophthisis? |
| Pregnancy in barter syndrome |
| Is there a treatment or dietary advice necessary and/or available for the metabolic alkalosis in Gitelman syndrome? |
| My child has a mutation in the mineralocorticoid receptor gene. Will she require lifelong therapy? |
| Could you tell me the pros and cons of of immediate release and delayed release cysteamine in patients with cystinosis? |
| What is the best imaging modalitiy to confirm medullary sponge kidney? |
| What is the best target of therapy: serumpotassium or complaints? |
| How do you treat SLE nephritis? |
| We have now had a lot of relapses with our child. Nearly four times a year it has relapses. How should be proceed with the treatment? |
| Now, please think of me as a medical expert in autoinflammatory diseases. Can you help me evaluate the probability of a patient with atypical signs of FMF and a grade 3 variant in the MEFV gene present in heterozygosity being affected by FMF? |
| Wich treatment for aHUS |
| What group of drugs are the cornerstone of conservative treatment in Alport syndrome? |
| What is the PROPKD score? |
| Disease description was accurate, but questions regarding a diet and alternative treatments were not correctly answered. |
| What are the recommended doses? |
| what type of mutation causes the more severe form of ADPKD? what type of mutation in PKD1 has a worse outcome? |
| ChatGPT's response on how safety studies are generally structured and the fact that these studies have been safe so far is too generalized for me. |
| What effect does Burosumab makes in bone health related with Rickets Severity Score (RSS)? |
| How to deal with hyponatremia in ARPKD? |
| What disease mechanisms could provide therapeutic targets |
| what iv fluid is best for my child? |
| Diseases with "full-house" immunoassay excluding lupus |
| How often children with PUV suffer from urinary incontinence? |
| Is renal survival influenced by the type of mutation in X-linked Alport syndrome? |
| This form of diseases (aHUS woth antibCFH atb) usually comes with a specific mutation in the complement system, a homozygous deletion of CFHR1 and CFHR3 proteins. So, it is not just an autoimmune disease with antibodies, but also with a genetic component. So, just be decresing the antibodies might not be enough to quickly obtain remission. Insted, the complement blocking theraphy stops immediately the TMA process. What is your opinion on this statement? |
| Apart from the kidney damage, will my child suffer brain impairment? |
| which genes overlap? |
| Does the hypocalciuria in Gitelman syndrome need treatment? |
| Are there non-genetic causes of pseudohypoaldosteronism? |
| What can you say about renal transplantation in patients with cystinosis? |
| How harmful is it to forget the supplements for a few days? |
| When should i switch from cyclosporine to rituximab based regimen? |
|  |
| **Unedited ePAG members emotional challenges for ChatGPT** |
| My baby has ARPKD should I terminate the pregnancy? Will he die after birth? What's the survival rate at birth? what's his life expectation? will he be disable for like? |
| What if i receive the wrong medicine? |
| I am so tired - what can I do? |
| What to do when my son has PUV and fever? |
| You told me to ask VSOP for more information but they are only an umbrella organisation and do not have specific information. What must I do now?? |
| My child is vomiting and tired, doesn't eat and drinks a lot. What to do? |

Supplemental Table 3:

| **Unedited comments submitted by ERKNet experts and ePAG representatives** | **Category** | **Rating** |
| --- | --- | --- |
| The possible downsides of genetic testing are not specifically addressed. There is however emphasis on genetic counseling, although I don't know whether every parent would understand what is meant there. | Advice on genetic testing | Neutral |
| recommended my centre, so clearly picked the best place :-) | Advice on second opinion or other medical centers | Positive |
| again, very generic and perhaps USA-centric ("Genetic testing can be expensive, and insurance coverage varies. It’s worth discussing with your healthcare provider and insurance company.") | Advice on second opinion or other medical centers | Negative |
| ERKNet and ESPN as well as ESPU are missing. Chat GPT suggest rather US organizations like Mayo Clinic, AUA, NKF (National Kidney Fundation). | Advice on second opinion or other medical centers | Negative |
| ERKNet does not appear in the results, also not GPN but "DGfN" and some American Information sites | Advice on second opinion or other medical centers | Negative |
| find a doctor answer refers first to adult centers, on third place is pediatric nephrology. The information source answer mixes up dutch and american sources | Advice on second opinion or other medical centers | Negative |
| In the case of the question: Where can I find a doctor for a second opinion close to [name of a familiar city]?....they only recommended pediatric endocrinologist specialists... | Advice on second opinion or other medical centers | Negative |
| misses important reference centers, does not mention ERKnet, neither well known patient's associations. Provides a reference center without any expertise in the disease | Advice on second opinion or other medical centers | Negative |
| neither www.neocyst.de nor www.erknet.gov appears as a reliable information source for NPH | Advice on second opinion or other medical centers | Negative |
| Only some - I don't know what way chosen centers for kidney issues are listed, not all localized the nearest. The information is not specific and without chat GPT looking for specialist would be easier and faster | Advice on second opinion or other medical centers | Negative |
| Surprisingly, my hospital is a reference center for genetic studies for kidney diseases in my country and is an ERKnet center and does not appear in CHATGPT as a suggestion. | Advice on second opinion or other medical centers | Negative |
| Very US-oriented (language, organization of healthcare, etc.) | Advice on second opinion or other medical centers | Negative |
| A lot of alternative treatments listed, however, a caution was advised and suggestion for a healthcare professionals consultation before starting any alternative treatments | Alternative treatments | Neutral |
| Chat-GPT said that there aren't any alternative treatments but said that some complementary approaches may offer additional support for example acupuncture, herbal supplements, mind-body practices etc. Help!! | Alternative treatments | Neutral |
| I dont know anything on complementary medicine. However they warn to always talk to healthcare provider if one wants to try complementary medicine and they explain there is no evidence for those therapies. However they give a lot of options that some parents might take as therapy options. | Alternative treatments | Neutral |
| alhtough it acknowledeges that seeking professional advice is required, it gives false hope with some alternative treatments | Alternative treatments | Negative |
| All possible alternative treatments are listed and at the end is statement that each should be consulted with healthcare provider. However I see some endanger connected with lack of very clear statemets that these therapies are really not checked for its safety. It should be more stressed! | Alternative treatments | Negative |
| CHATGPT says: Herbal Supplements: Astragalus: May help support kidney function and reduce proteinuria. However, clinical evidence is limited, and it should be used with caution. Cordyceps: A type of mushroom that might have kidney-protective properties, though more research is needed. | Alternative treatments | Negative |
| Here it starts getting unfunded and potentially harmful. ChatGPT addresses the possibilities of herbal medicines, acupuncture and homeopathy. While it says that these treatments are of not proven, they are being presented as logical alternatives. | Alternative treatments | Negative |
| Recommends ginger and liquorice! As well as any other quackery around, such as "Mind-body-techniqiues". Not sure, homeopathy and acupuncture are going to be very helpful either....This seemed to be totally unconnected to NDI and more about alternative remedies in general. | Alternative treatments | Negative |
| This response of ChatGPT is full of general information regarding complementary medicine (with indeed fair and to a large extent correct information ("some families believe...", but no evidence)) My personal fear is that one might believe that since ChatGPT gives a credible response, one might think along the lines of "where there is smoke, there is a fire!" and thus conclude that complementary therapies do have a place in treating PHA. | Alternative treatments | Negative |
| Although this question was more emotional, reflecting parents worries, the answer was very similar to previous one that was more informative and providing facts. Nevertheless, the answer did provide some reassurance and even empathy. | Empathy | Positive |
| I am a bit surprised. The answers are way much better than I thought they would be. | General | Positive |
| perfect | General | Positive |
| The answers are more complete than those we can give in a simple query. | Information depth of responses | Positive |
| ChatGPT provided a clear and readable statements | Language | Positive |
| The information provided by ChatGPT is easily readable, but I think that is still too difficult for the average-educated reader. It is fairly accurate, but misses some aspects that are quite relevant to a parent (prognosis? severity? more detailed information about treatment? | Language | Neutral |
| The prompt id crucial. Some of the information provided can mix technical and not technical language | Language | Neutral |
| The language in the answer may be to difficult as first information for many people | Language | Negative |
| They don´t talk anything about new treatments like Burosumab, for example (they said that there aren´t any other treatments) | New therapies | Negative |
| It is important that almost every hint given by ChatGPT is ended up with suggestion of contact with healthcare provider. | Safety | Positive |
| The Chat-GPT constantly emphasizes the importance of communicating with the responsible medical team, which is excellent | Safety | Positive |
| Generalizations always have a risk. There is a disclaimer in the text ChatGPT delivers about that. | Safety | Neutral |
| again, very generic and the following ("Recognize the signs of dehydration, such as dry mouth, sunken eyes, or decreased urination, and seek medical attention if they occur.") is really not appropriate for NDI:if they wait till the child has decreased urination, it is way too late. | Safety | Negative |
| chat gpt cannot know the exact situation of the child, for not knowing it the suggestions are quite good | Specificity of responses | Positive |
| again, superficial but acurate and for every answer they tell you to talk to the healthcare professional. Its ok... | Specificity of responses | Neutral |
| Although the information given remains somewhat superficial/vague, the main message is still accurate | Specificity of responses | Neutral |
| chat gpt skips the very severe cases and gives a lot of hope | Specificity of responses | Neutral |
| ChatGPT only mentions the NTx, not the LTx/NTx, but the basic information is good (e.g., Tx is not a cure, treatment via FA). ChatGPT, for example, mentions the OHF (Oxalosis & Hyperoxaluria Foundation as a patient organization). | Specificity of responses | Neutral |
| ChatGPTprovides very general and basic answers, for example it says that the treatment is for blood pressure and proteinuria, but does not specify which ones. | Specificity of responses | Neutral |
| it simple but all true and not too alarming. However the disease has a large spectrum wich of course is not addressed | Specificity of responses | Neutral |
| The answer is quite superficial but appropriate | Specificity of responses | Neutral |
| The chat-GPT was correct but provided relatively limited information | Specificity of responses | Neutral |
| The explanations of the diet are focused on when there is advanced chronic kidney disease. | Specificity of responses | Neutral |
| again to vague and mixing lay with medical language | Specificity of responses | Negative |
| Chat GPT suggested that only urolgist is in charge: "...Regular follow-ups with a urologist to monitor kidney and bladder function..." ChatGPT provide information that:...Early detection and treatment are crucial to manage the condition and prevent serious complications..." which is not true in some cases. | Specificity of responses | Negative |
| For this particular disease, a high salt intake is advised. ChatGPT talks of a "slightly increased salt intake" which is very mildly put. Patients have hypocalcemia with tendency towards hypercalcemia, especially when other triggers for hypercalcemia are present. Contraintuitively, ChatGPT suggests to supplement calcium and vitamin D for deficiency and bone health, which is contraproductive and not necessary. | Specificity of responses | Negative |
| I copy-pasted the questions in English, so ChatGPT comes up with info in English. The Dutch patient information for this disorder (therefore?) is not displayed. | Specificity of responses | Negative |
| Response: "Avoiding Caffeine and Sugary Drinks: Why: Caffeine and sugary drinks can increase urine production. How: Limit or avoid beverages like soda, caffeinated tea, and coffee." a) there is no evidence that caffein enhances urine output further in NDI and b) sugary drinks are exactly the kind of supplements we like to use in NDI to ensure sufficient caloric intake! There is also nonsense about potassium rich diet and bones: "Potassium-Rich Foods: Why: Potassium helps maintain fluid balance and can be beneficial in managing NDI. How: Include foods like bananas, oranges, potatoes, and spinach. Calcium and Vitamin D: Why: Some children with NDI may have issues with bone density. How: Ensure adequate intake of calcium and vitamin D through dairy products, fortified foods, or supplements if recommended by a healthcare provider." | Specificity of responses | Negative |
| Some advices refer to kidney stones in general. | Specificity of responses | Negative |
| Too many general recommendations, some only apply for severe kidney damage or CKD. | Specificity of responses | Negative |
| Very generic answer. Uses terms such "reabsorption", which is probably meaningless or even confusing without explanation. | Specificity of responses | Negative |

Supplemental Figure 1: Origin of participating ERKNet experts and ePAG representatives


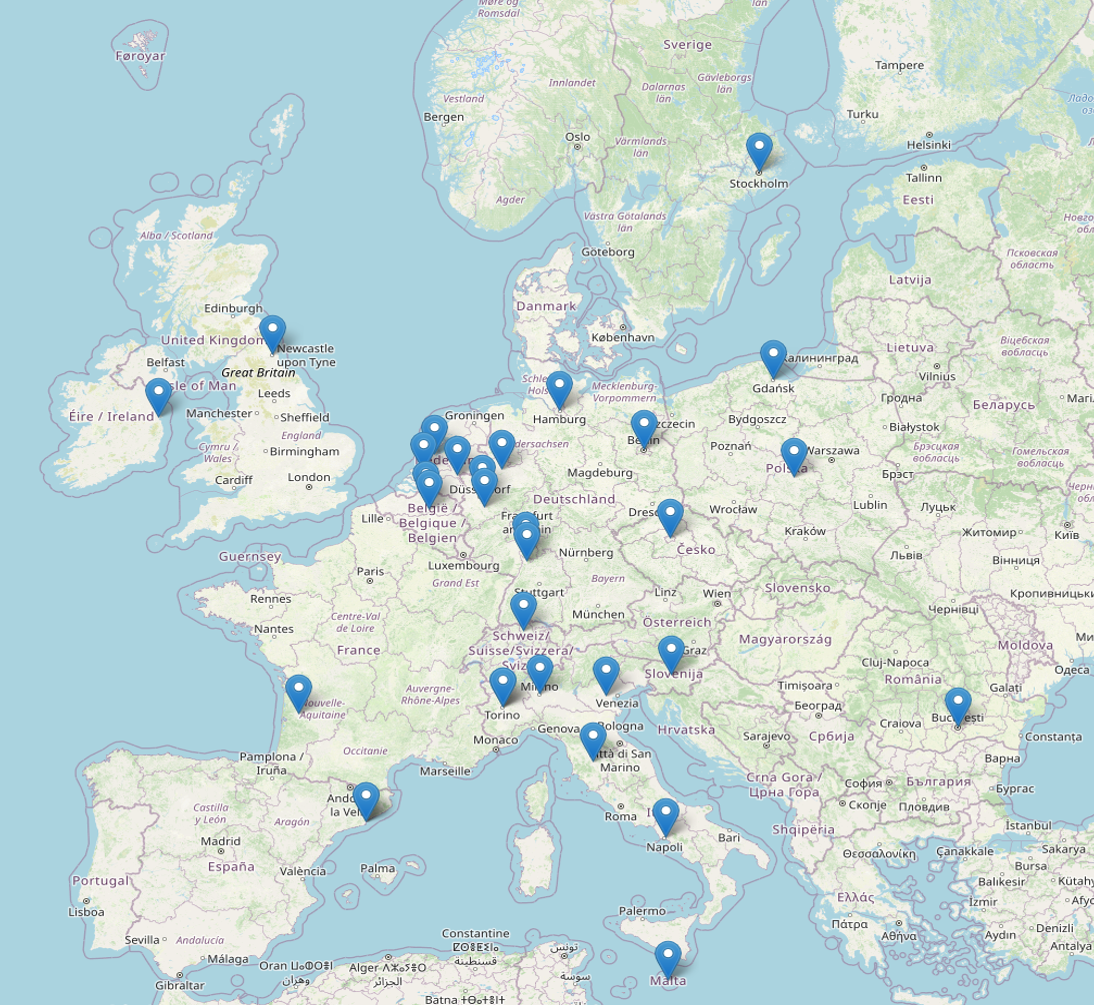


Source: Open Street Map (<https://www.openstreetmap.org/>)
